# The Global Consortium for Drug-resistant Tuberculosis Diagnostics (GCDD): design of a multi-site, head-to-head study of three rapid tests to detect extensively drug-resistant tuberculosis

**DOI:** 10.1186/1745-6215-15-434

**Published:** 2014-11-06

**Authors:** Naomi Hillery, Erik J Groessl, Andre Trollip, Donald Catanzaro, Lynn Jackson, Timothy C Rodwell, Richard S Garfein, S-Y Grace Lin, Kathleen Eisenach, Theodore G Ganiats, Daniel Park, Faramarz Valafar, Camilla Rodrigues, Valeriu Crudu, Thomas C Victor, Antonino Catanzaro

**Affiliations:** Department of Family & Preventive Medicine, University of California, San Diego, CA USA; Department of Biomedical Sciences, Stellenbosch University, Cape Town, South Africa; Division of Bioinformatics and Medical Informatics, San Diego State University, San Diego, CA USA; Department of Medicine, University of California, San Diego, CA USA; California Department of Public Health, Richmond, CA USA; Department of Pathology, University of Arkansas for Medical Sciences, Little Rock, AR USA; Hinduja National Hospital, Mumbai, India; Microbiology and Morphology Laboratory, Institute of Phthisiopneumology, Chisinau, Moldova

**Keywords:** drug resistance, international, multi-site, rapid diagnostic tools, tuberculosis, XDR-TB

## Abstract

**Background:**

Drug-resistant tuberculosis (DR-TB) remains a threat to global public health, owing to the complexity and delay of diagnosis and treatment. The Global Consortium for Drug-resistant Tuberculosis Diagnostics (GCDD) was formed to develop and evaluate assays designed to rapidly detect DR-TB, so that appropriate treatment might begin more quickly. This paper describes the methodology employed in a prospective cohort study for head-to-head assessment of three different rapid diagnostic tools.

**Methods:**

Subjects at risk of DR-TB were enrolled from three countries. Data were gathered from a combination of patient interviews, chart reviews, and laboratory testing from each site’s reference laboratory. The primary outcome of interest was reduction in time from specimen arrival in the laboratory to results of rapid drug susceptibility tests, as compared with current standard mycobacterial growth indicator tube (MGIT) drug susceptibility tests.

**Results:**

Successful implementation of the trial in diverse multinational populations is explained, in addition to challenges encountered and recommendations for future studies with similar aims or populations.

**Conclusions:**

The GCDD study was a head-to-head study of multiple rapid diagnostic assays aimed at improving accuracy and precision of diagnostics and reducing overall time to detection of DR-TB. By conducting a large prospective study, which captured epidemiological, clinical, and biological data, we have produced a high-quality unique dataset, which will be beneficial for analyzing study aims as well as answering future DR-TB research questions. Reduction in detection time for XDR-TB would be a major public health success as it would allow for improved treatment and more successful patient outcomes. Executing successful trials is critical in assessment of these reductions in highly variable populations.

**Trial registration:**

ClinicalTrials.gov NCT02170441.

## Background

Tuberculosis (TB) is among the top threats to global public health, ranked second only to HIV as the most deadly infectious disease worldwide by the World Health Organization (WHO) [1]. Achievements in TB control over the last decade have led to a slow decline in TB incidence of 2% per year [1]; however, drug-resistant TB (DR-TB) remains a serious public health concern globally. The WHO estimated that in 2012 there were 450,000 new multidrug-resistant TB (MDR-TB) cases; that is, cases of TB resistant to first-line drugs isoniazid and rifampin. Additionally, an estimated 9.6% of MDR-TB cases were also extensively drug-resistant (XDR-TB), having additional resistance to fluoroquinolones, such as ofloxacin and moxifloxacin, and at least one of the injectable anti-TB drugs (capreomycin, kanamycin, and amikacin), making treatment virtually impossible in countries without access to alternative drugs.

Critically, the WHO reported that fewer than 25% of the 450,000 estimated MDR-TB cases in 2012 were actually detected [1]. Drug-resistant TB can be managed successfully only if it is diagnosed rapidly using drug susceptibility testing, allowing for prompt and appropriate treatment. Standard, culture-based phenotypic drug susceptibility tests take weeks to months for results, which delays treatment and can significantly impact TB treatment outcomes [2]. Delayed patient management decisions contribute to amplification of resistance, transmission of DR-TB to uninfected individuals, and higher mortality rates. Studies have shown that if TB is correctly diagnosed and promptly treated, transmission to uninfected individuals is quickly reduced; however, if diagnosis and treatment are delayed or if TB is not detected, spread of DR-TB is likely [3, 4].

In 2008, the United States National Institutes of Health placed the development and testing of technologies for rapid drug resistance detection at the top of their list of TB research priorities [5]. The Global Consortium for Drug-resistant Tuberculosis Diagnostics (GCDD) was established in 2008 to characterize the genetic basis of drug resistance and evaluate molecular and microbiological methods of detecting DR-TB quickly and efficiently. This international collaboration to improve current DR-TB diagnostics gathered data from three unique regions in an effort to improve accuracy and precision of novel diagnostics and reduce DR-TB detection time. Here we explain the methods employed by GCDD investigators to conduct a multinational longitudinal cohort study that addresses these objectives.

### Aims and hypotheses

Study aims were defined as follows in the approved protocol of the National Institute of Allergy and Infectious Diseases Division of Microbiology and Infectious Diseases (text edited for clarity and brevity):

Aim 1: To reduce the average XDR-TB detection time from months to a week. We will compare the performance of one existing line probe assay (Hain GenoType®MTBDR*plus* and Hain GenoType®MTBDR*sl*) with a newly developed sequence-based assay (pyrosequencing) and an expanded Microscopic Observation Drug Susceptibility (MODS) phenotypic assay, to detect resistance to isoniazid and rifampin, ofloxacin, moxifloxacin, kanamycin, amikacin, and capreomycin.

Aim 2: To determine agreement between rapid tests and standard drug susceptibility test results. Results of rapid tests based on smear-positive sputum will be compared with those from the current gold-standard drug susceptibility test method, which is based on subculture of *Mycobacterium tuberculosis* growth in the MGIT 960 liquid culture system.

Aim 3: To identify the genetic basis of discordant results from Aim 2. Strains identified as drug resistant by the drug susceptibility test, but not by the gene-based molecular tests, will be further examined through sequencing target genes, neighboring genes, or the entire genomes of unique strain families.

Aim 4: To characterize XDR-TB strains globally. Genotypic, phenotypic, and epidemiological features, as well as geographical relationships of XDR-TB strains, will be characterized and compared with other drug-resistant and susceptible strains. We will explore the relationships between drug resistance, including XDR-TB, and patient risk factors and strain families of *M. tuberculosis*.

#### Secondary aims

Secondary Aim 1: Cost-effectiveness study. The costs associated with performing each rapid test will be compared with improvements in time and accuracy for detecting drug resistance and XDR-TB over standard drug susceptibility testing methods.

Secondary Aim 2: To determine the predictive value of resistance-associated mutations in determining sputum culture conversion.

## Methods

The GCDD study design was conducted in two phases. Phase I involved the creation of a specimen repository by requesting banked isolates of *M. tuberculosis* from TB centers in four geographically distinct sites that included Manila, the Philippines; Mumbai, India; Chisinau, Moldova; and Port Elizabeth, South Africa. The isolates requested were selected to maximize phenotypic and genotypic diversity in the repository, which would be used to inform the design and evaluate the performance of molecular assays for detecting drug-resistant TB. Upon receipt, all repository isolates were genotyped using 12 mycobacterial interspersed repetitive units, spoligotyping, and Sanger sequencing. Drug susceptibility tests were also performed using MGIT 960 according to the manufacturer’s instructions. Additional methods and results of this study have been described elsewhere [6]. Data from this repository were used to inform the design of diagnostic tests that were evaluated in the second phase.

Phase II, which is the focus of this paper, consisted of a prospective cohort study designed to evaluate a number of rapid drug susceptibility tests compared with the standard MGIT 960 assay among patients with suspected, but not confirmed, XDR-TB in existing clinical laboratories in three of the countries included in Phase I. Participants were screened for eligibility and provided written informed consent prior to enrollment. Biological specimens and patient interview data were collected at baseline and 52-week follow-up visits. In addition, medical record reviews were conducted at baseline, 30 days post-enrollment and 52 weeks post-enrollment. Sputum specimens collected were tested using MGIT drug susceptibility tests, line probe assay, pyrosequencing, and MODS assay. In addition to the results of a rigorously standardized, direct comparison of the performance of these assays, this study produced a well-characterized repository of *M. tuberculosis* isolates to address many other clinical, laboratory and epidemiologic questions. This paper describes the methodology used for the prospective cohort study.

### Study sites

Participants were enrolled from three diverse regions with a high prevalence of XDR-TB: Mumbai, India; Chisinau, Moldova; and Port Elizabeth, South Africa. These sites were carefully considered when planning the study and were selected because of a high documented risk of DR-TB and the ethnic diversity of these regions.

#### India

The PD Hinduja National Hospital and Medical Research Centre is a tertiary care center in central Mumbai, India that provides medical care in all specialties of Medicine and Surgery. The Pulmonary Department at the PD Hinduja National Hospital is the busiest in Mumbai and is the referral center for MDR and XDR-TB cases of the city and the state of Maharashtra. Therefore, the TB patient population is more likely to contain those who have previously been treated and were either unresponsive or relapsed [7]. In a consecutive sampling of 150 patients in the Mumbai area, 80% of samples obtained were found to be resistant to one or more standard TB medications, while 51% were resistant to more than one drug [7].

#### Moldova

The Phthisiopneumology Institute in Chisinau, Moldova, the central unit of the Moldovan National TB Control Programme, is a scientific research, medical consultation, and training center, which leads all TB and unspecific upper respiratory tract diseases services for patients across Moldova. Disintegration of the Soviet Union in the early 1990s resulted in a sudden and sharp deterioration of socioeconomic conditions in Moldova, leading to an upsurge in TB from increased infection and increased risk of breakdown from infection to disease. A financial crisis led to drug shortages and inadequate and interrupted treatment. As a result, drug resistance increased with the eventual emergence and transmission of MDR strains. The prevalence of MDR-TB in Moldova has been documented as 24% of new and 62% of previously treated patients, according to national TB surveillance data between 2007 and 2010 [8].

#### South Africa

South Africa is one of the five countries with the largest number of incident TB cases in 2011, estimated to be between 0.4 million and 0.6 million, according to the WHO. Co-infection with HIV is of particular concern in this region, with 65% of patients with TB known to be HIV-positive [9]. In Port Elizabeth, patients were enrolled at six Primary Health Care facilities and one regional hospital. Decentralized enrollment resulted in a different prevalence of drug resistance at this site.

### Eligibility

Newly presenting patients with TB who were 5 years of age and older, and patients for whom treatment failed, were recruited from each of the study clinics. Owing to varying circumstances and constraints affecting recruitment at each site, local recruitment procedures were documented in site-specific standard operating procedures and sent to the study coordinators prior to enrollment. For example, the frequency of recruitment was expected to vary among the study sites to accommodate differences in clinical staffing of the study, enrollment locations, and laboratory capacity. All site-specific standard operating procedures followed the same general guidelines set forth in the GCDD study clinical protocol. Study protocols were approved by the institutional review board of the University of California, San Diego (Project No. 1100383) and by the institutional review board of each enrolling site: PD Hinduja National Hospital and Medical Research Centre, Project Number. 507-09-CR; the Ministry of Health Care of the Republic of Moldova, Institution of Public Health, and Ethics Committee of the Phthisiopneumology Institute of Moldova (no applicable reference number); and Universiteit-Stellenbosch University Health Research Ethics Committee Tygerberg, South Africa, ethics reference number N10/08/261.

### GCDD study eligibility criteria

Participants were included in the study if they

- Were at least 5 years of age
- Were acid-fast bacilli sputum smear-positive , 1+ or greater (within previous 14 days), positive on GeneXpert, or present clinically with high suspicion of active TB **and**:
  - Previously received >1 month of treatment for a prior TB episode **or**
  - Were failing TB treatment with positive sputum smear or culture after ≥3 months of a standard TB treatment **or**
  - Had close contact with a known drug-resistant TB case **or**
  - Were newly diagnosed with MDR-TB within the last 30 days **or**
  - Were previously diagnosed with MDR-TB and failed TB treatment with positive sputum smear or culture after ≥3 months of a standard MDR-TB treatment regimen
- Provided informed consent or subject or legal guardian or representative able and willing to provide informed consent

Participants were excluded from the study if they

- Were institutionalized
- Were unable to provide at least 7.5 ml sputum (1st and 2nd samples combined)
- Had results from Second-line drug susceptibility test performed within the last 3 months

The eligibility criteria for this study were designed to identify patients at increased risk of DR-TB. Potential study subjects gave oral consent to be screened for study eligibility. The screening was performed by a trained clinical staff member using a computerized algorithm during a single study visit. Written consent, or assent if the patient was younger than 18 years old, was collected if eligibility was confirmed. Children between the ages of 5 and 17 were eligible for the study at sites where the participation of minors was approved by the local institutional review board or independent ethics committee. Subjects were not compensated for participation; however, in India and South Africa where patients traveled an hour or more from their residence for study visits not related to routine care, travel costs were reimbursed.

### Participant withdrawal

Enrolled subjects were withdrawn from the study if they could not provide adequate sputum for testing. Participants could also request to be withdrawn from the study. If this occurred, the reasons were documented. Once participants were withdrawn, their study data were excluded from all analyses. Failure to return for follow-up or learning of a participant’s death were not considered reasons for withdrawal from this study.

### Training

Prior to study initiation, all personnel gathered in San Diego, California, USA for training in good clinical practices and the GCDD protocol. The training in good clinical practices was conducted by Family Health International, contracted by the National Institutes of Health and the National Institute of Allergy and Infectious Diseases, so that scientists were certified in ethical considerations with human subjects and international clinical trial protocols. All domestic and international collaborators also completed certification in the Collaborative Institutional Training Initiative.

Clinical and laboratory personnel from each site were also trained in the use of case report forms and study laptops, used for clinical data collection. Laboratory technicians specializing in the pyrosequencing assay were given detailed training at the California Department of Public Health in Richmond, California, while those receiving detailed training in the MODS assay were trained in Lima, Peru. Site personnel were generally more experienced in the use of line probe assays, so study procedures were reviewed at the meeting in San Diego. As procedures for the study were refined or new elements added, follow-up training was conducted via webinar. These were run by the staff most knowledgeable in the procedures and were targeted to certain collaborators at each site. For example, updates to the clinical case report forms were presented by the data manager to the enrolling clinicians. Principal investigators were invited to attend but were not required.

### Enrollment procedures

Study procedures were complex and several clinical and laboratory processes needed to be standardized across the distinctive sites. Therefore, we included a series of validation procedures, which utilized enrolled subjects’ samples to validate all data systems and to allow laboratories time to become proficient in all assays and procedures. Completion of validation procedures was a requirement to initiate the study.

Following screening and informed consent procedures, eligible patients were asked to provide a spot sputum specimen and complete a baseline interview. Patients were asked to return to the clinic the following day with a sputum specimen produced upon waking in addition to a second spot sputum at the clinic. At 52 weeks after enrollment, subjects were asked to return one final time to provide a single spot sputum specimen and complete a brief clinical examination. With the subject’s consent, medical record reviews were conducted at baseline, day 30, and week 52 to provide supplementary information to that obtained during patient interviews and to document detailed treatment history for the TB illness diagnosed at the time of study enrollment. Sputum specimens collected at each visit were sent to the central site laboratories to be processed for testing. Upon completion, culture isolates were shipped to the University of California, San Diego, for storage.

### Case report forms

Sources for the data collected for the study included patient interviews, chart reviews, and laboratory processing information. Standardized case report forms developed by researchers at the University of California, San Diego, gathered all clinical and laboratory processing information. Table 1 lists all study case report forms and samples, and the times at which each were collected.

**Table 1:** **Data collection schedule**

Tests, measures, and samples	Clinic or laboratory	Baseline	Day 2	30 days	52 weeks
Sputum sample	Clinic	×	×		×
Blood sample (some study sites)	Clinic	×			
Screening interview	Clinic	×			
Enrollment interview	Clinic	×			
Enrollment chart review	Clinic	×			
Day-2 specimen collection	Clinic		×		
30-day chart review	Clinic			×	
52-week interview	Clinic				×
52-week chart review	Clinic				×
Sample collection	Laboratory	×			×
Sample processing	Laboratory	×			×
Smear microscopy	Laboratory	×			×
Mycobacterial growth indicator tube culture	Laboratory	×			×
Presumptive identification	Laboratory	×			×
Confirmatory identification	Laboratory	×			×
Löwenstein-Jensen culture	Laboratory	×			×
Mycobacterial growth indicator tube drug susceptibility test	Laboratory	×			×
Hain GenoType®MTBDR*plus*	Laboratory	×			
Hain GenoType®MTBDR*sl*	Laboratory	×			
Microscopic observation drug susceptibility assay	Laboratory	×			
Pyrosequencing	Laboratory	×			
Freezing and storage	Laboratory	×			×

#### Interviews

The baseline interview gathered demographic measures such as age, race, ethnicity, and sex. Known TB risk factors were assessed at baseline and for the previous three months. Maps were given to each enrolling clinician so that detailed geographical history could be assessed; this was used for spatial analyses. A clinical history was obtained at this visit, including current symptoms, co-occurring conditions, HIV or AIDS status, height, and weight. The follow-up interview was less detailed and assessed subject’s treatment status, current symptoms, height, and weight.

#### Chart reviews

The baseline chart review included measures of the subject’s current TB category (new or previously treated), TB drug history beginning from diagnosis of the current illness, history of chest X-ray and results, and HIV status. If HIV-positive, CD4 counts and viral loads were requested. The 30-day chart review documented the subject’s current status, TB drug history beginning from diagnosis of the current illness and, if unavailable at baseline, HIV status. The 52-week chart review assessed the subject’s current status, TB drug history beginning from diagnosis of the current illness, history of smear and culture performed since study entry, and results of the most recent chest X-ray, if performed.

#### Laboratory data collection

Using the standard laboratory case report form, laboratories recorded the date and time of the collection and receipt of the sputum samples. Information on the quality and quantity of the sputum was also recorded. Sputum processing was performed using the standard N-acetyl-L-cysteine sodium hydroxide method [10]. The date and time that cultures were started and completed were recorded, and later used to calculate the culture time to detection. Culture and MGIT drug susceptibility test results were transcribed from the MGIT 960 instrument, while standardized worksheets were used to record observed data from the MODS assay. Line probe assay results were recorded directly onto laboratory case report forms. The line probe assay strips were scanned for later verification. Pyrosequencing data were transcribed onto the laboratory case report form from the Pyromark Q96 instrument after comparison with the Pyrosequencing Library [11]. Pyrograms were saved as.pdf and uploaded to the website for further analysis. Owing to standard batching procedures, results for several patients could appear in test results. This transfer of information allowed data for a subject coming from several sources to be gathered in one location.

### Reference standard and rapid tests

The reference standard for phenotypic observation of drug susceptibility test used in this study was the MGIT 960 TB system. The study drugs described in Aim 1, as well as isoniazid and rifampin, were tested using a MGIT drug susceptibility test when the culture tested positive, while sputa from each subject were tested with the three rapid tests regardless of culture status. Procedures were as follows.

#### Mycobacterial growth indicator tube drug susceptibility test

In MGIT drug susceptibility testing, the drug susceptibility of TB is based on the modified proportion method (Food and Drug Administration approved for first-line anti-TB drugs). The critical proportion for resistance is taken as 1% for all anti-TB drugs, meaning that if 1% or more of the test mycobacterial population is resistant, the culture is considered resistant. We determined resistance by comparing growth in MGIT tubes with and without drugs; this was accomplished in an instrument with the capacity to monitor growth of 960 MGIT culture tubes simultaneously. Although the first-line drugs were available from Becton Dickinson, the second-line drugs were not. Thus, testing of second-line drugs was performed using validated critical concentrations of in-house (locally prepared by each site) drug solutions compatible with the WHO recommendations: 2.0 μg/ml for ofloxacin, 0.25 μg/ml for moxifloxacin, 1.0 μg/ml for amikacin, and 2.0 μg/ml for capreomycin [12]. As there were no published WHO recommended critical concentrations for kanamycin drug susceptibility testing by MGIT 960 at the time of the study, we used 2.5 μg/ml, based concentrations reported in the literature [13, 14].

#### Line probe assays

Commercial line probe assays, such as the GenoType®MTBDR*plus* (Hain Lifesciences, Nehren, Germany), have been well validated for the detection of isoniazid and rifampin resistance indirectly using TB culture isolates and directly on smear-positive clinical specimens. Barnard *et al.* conducted a large study using GenoType®MTBDR*plus* on smear-positive sputum specimens from 536 patients at a high risk of MDR-TB [15]. They found a sensitivity and specificity of 98.9% and 99.4%, respectively, for detection of rifampin resistance; sensitivity was 94.2% with specificity of 99.7% for detection of isoniazid resistance. Results were interpretable for 97% of the specimens within 1 or 2 days. Overall, these studies demonstrate that the strip assay is rapid and accurate for the detection of mutations found in MDR-TB strains, providing an excellent platform for development to detect XDR-TB strains. The GenoType®MTBDR*sl* strip is marketed as an indirect test but is often used to test specimens directly for resistance to second-line drugs. Few studies have been published that evaluate the performance of the GenoType®MTBDR*sl* test to detect resistance to second-line drugs in *M. tuberculosis* isolates and sputum specimens. Brossier *et al.* reported sensitivities and specificities of the MTBDR*sl* test in a study of 49 clinical isolates (41 MDR-TB and 8 XDR-TB) as follows: fluoroquinolones 87%, 96%; amikacin 100%, 100%; kanamycin 77%, 100%; and capreomycin 80%, 98% [16]. Kiet *et al.*, in a study of 41 fluoroquinolone resistance isolates and 21 MDR-TB but fluoroquinolone-sensitive isolates, reported sensitivities and specificities of the MTBDR*sl* test as follows: fluoroquinolones 75.6%, 100%; kanamycin 100%, 100% [17]. These studies demonstrate that, because of mutations not included in the test (such as *gyr*B), or unknown resistance mechanisms, the GenoType®MTBDR*sl* tests lacks the sensitivity to exclude the possibility of second-line drug resistance reliably. Both GenoType®MTBDR*plus* and GenoType®MTBDR*sl* were evaluated in the clinical observation testing phase.

These assays are based on the principle of amplification of DNA isolated directly from decontaminated patient specimens followed by hybridization with specific membrane-bound probes using a primer-nucleotide mix. The first step of the procedure was isolation of mycobacterial DNA from a decontaminated patient specimen by a heat and sonication method. The second step involved a multiplex amplification with biotinylated primers in a thermal cycler. A third step involved chemical denaturation of the amplification products and hybridization of the single-stranded, biotin-labeled amplicons to membrane-bound probes. Finally, a streptavidin-alkaline phosphatase conjugate was added to initiate an alkaline phosphatase staining reaction. Each of the reference laboratories had a thermal cycler suitable for the amplification step. Banding pattern results were interpreted using the template provided with the kit.

#### Pyrosequencing

Pyrosequencing is a sequence-based molecular method to detect mutations associated with drug resistance. The method requires DNA extraction, amplification by PCR, and real-time sequencing, using pyrosequencing technology and the PyroMarkQ96 platform, as described previously [11, 18]. The GCDD methods for pyrosequencing were guided by a study conducted by Bravo *et al.*, in which 102 clinical *M. tuberculosis* isolates were evaluated for susceptibility to rifampin, isoniazid, and ofloxacin with a pyrosequencing assay. The sensitivities and specificities of the assay were 96.7% and 97.3%; 63.8% and 100%; and 70.0% and 100% for the detection of resistance to rifampin, isoniazid, and ofloxacin, respectively [19]. In this study, we included eight molecular targets: IS6110, for identification of *M. tuberculosis*, *katG*, the *inhA* promoter, and *ahpC* for isoniazid, *rpoB* for rifampin, *gyrA* for quinolones, and *rrs* for injectable drugs.

Pyrosequencing was used to rapidly characterize mutations within specific genes associated with resistance to isoniazid, rifampin, the fluoroquinolones, and the injectable drugs. Pyrosequencing was performed according to the manufacturer’s instructions using the sequence analysis mode of the Pyromark 96 ID system (Qiagen, Valencia, CA, USA) and the standard Pyromark Gold Q96 reagent kit, containing enzyme, substrate, and nucleotides (Qiagen, Valencia, CA, USA). Briefly, biotin-labeled PCR products were immobilized on streptavidin-coated sepharose beads and denatured to serve as single-stranded DNA templates. These beads were subsequently transferred to a 96-well plate containing annealing solution and sequencing primer. The reaction cascade primarily consists of the incorporation of nucleotides into the growing DNA chain, culminating in the production of light. The pattern of emitted light in relation to the nucleotide dispensation order and number of nucleotides incorporated was subsequently illustrated on a pyrogram. The data were analyzed using IdentiFire software, supplied by the manufacturer. Susceptibility results based on genotypic testing were compared using a phenotypic drug susceptibility test.

#### Microscopic observation drug susceptibility assay

The MODS assay is a well-described microbiological technique that has shown to be reliable, cheap, and easy to implement in low-volume, low-resource settings for drug susceptibility testing. The performance of the MODS assay in identification of MDR-TB is excellent, with a sensitivity of 97.8% and a specificity of 99.6%, compared with standard drug susceptibility testing methods [20]. The rapidity, simplicity, and low cost of the MODS assay made it a promising candidate for use as a methodology for second-line drug resistance testing and XDR-TB detection. Although the MODS assay has been validated for detection of resistance to first-line drugs, a reliable methodology for detection of resistance to ofloxacin, amikacin, kanamycin, and capreomycin has not yet been established. The development of the MODS assay for the detection of second-line drug resistance has recently been described [21].

The MODS assay is a microscope-based assay that exploits the fact that *M. tuberculosis* grows more rapidly in liquid broth than solid medium and forms specific ‘cord’ formations that can be seen through the microscope long before colonies on solid media are visible to the naked eye. The MODS assay method uses a 24-well culture plate format. Patient sputum samples were digested and decontaminated according to a standardized procedure and inoculated into culture broth with and without the study drugs. The broth was then placed in the culture plates. If the sample of *M. tuberculosis* grew in broth alone, but not in drug-containing wells, if was drug-sensitive. If *M. tuberculosis* also grew in drug-containing wells, DR-TB was present. An advantage of the MODS assay is its flexibility to test multiple drugs at different concentrations at once. When used directly to detect *M. tuberculosis* in sputum, results are available in as early as seven days instead of two to three weeks following positive culture [20].

### Sample size and power analysis

The primary outcome measure for Aim 1 was the time to obtaining a drug susceptibility test result from the new tests, in comparison with the approximate 21 days that it typically takes to obtain this information using the standard methodology of MGIT culture and indirect drug susceptibility testing. Based on preliminary data of the novel diagnostic tests and the scientific methodology, study investigators were confident that the time to result would be shortened dramatically. Prior to study launch, power analyses were conducted for each study aim and the ideal sample size was determined using PASS software (Number Cruncher Statistical Systems, Kaysville, UT, USA, version 2005). This software was used to calculate the power to conduct a two-sided paired *t* test, assuming a significance level of 0.05, a null-hypothesis difference of 0 day, an expected difference of 14 or 7 days, and an estimated standard deviation of 14 days. In a study with 100 patients with XDR-TB, we would have 100% or 99.9% power to find a significant reduction of 14 or 7 days, respectively. With as few as 10 patients having XDR-TB in a site, we would have 80.3% power to find a significant reduction of 14 days. This power analysis focused on XDR-TB, the group with the smallest and most restrictive sample size.

The goal of Aim 2 was to examine the agreement of the new tests with MGIT drug susceptibility tests for detecting the resistance of samples to first- and second-line medications. Here, the size of the confidence intervals around the sensitivity and specificity for each diagnostic test was a function of sample size and prevalence of resistance. The prevalence of resistance varied quite widely across the seven drugs examined, as well as across the algorithms for diagnosing MDR-TB and XDR-TB. To be conservative and align with the stated goals of the study, we used an estimated prevalence for XDR-TB, since its prevalence is much lower than MDR-TB. We estimated that the prevalence of XDR-TB would be 5% to 10% for the study. Based on preliminary study results, we estimated that the new tests would achieve a sensitivity of 0.90 to 0.95 and a specificity of 0.95 to 0.98. There is no standard on the ideal size of a confidence interval, simply smaller is better, but huge sample sizes may be required to accomplish this when detecting less prevalent cases. In addition, as prevalence decreases, it is easier to be confident about high specificity while harder to keep sensitivity high. Therefore, we estimated the sample sizes required to achieve confidence intervals of about 0.100 for sensitivity and 0.020 for specificity. It was determined that a sample size of 1,225 subjects would provide a confidence interval of 0.077 for a sensitivity of 0.95 and 0.017 for a specificity of 0.98 at an XDR-TB prevalence of 10%. If the prevalence of XDR-TB were as low as 5%, 1,225 subjects would provide a confidence interval of 0.105 for a sensitivity of 0.95 and 0.016 for a specificity of 0.98. This same sample size produces much tighter confidence intervals for MDR-TB, where prevalence is higher. At 35% MDR-TB prevalence, we obtain confidence intervals of 0.041 for a sensitivity of 0.95 and 0.018 for a specificity of 0.98.

Aim 3 (to identify the genetic basis of discordant results from Aim 2) was a developmental aim, and did not test specific hypotheses. Aim 4 (to characterize XDR-TB strains globally) was a descriptive analysis. It also did not test specific hypotheses. We expected a sample size of 1,225 to provide more than enough data to explore discordant results with genetic sequencing and characterize global strains using genotypic, phenotypic, epidemiological, and geographical variables.

The study did not randomize patients in the clinical data collection phase because it would have severely limited the statistical power of the confidence intervals for sensitivity and specificity calculations, and compromised our ability to complete the study. Instead, we chose to conduct all four resistance tests on every sample, allowing approximately 900 to 1,000 comparisons for each test. If patients were randomized to receive only one test, the study would have had to enroll over 4,000 patients to achieve the same confidence in our calculations.

### Study management

#### Organization

The study operated under a centralized study management group of administrators and laboratory technicians at the University of California, San Diego, under the supervision of the principal investigator, Dr. Antonino Catanzaro. Several committees existed within the study framework so that co-investigators could provide key advice as the study progressed. Working groups who met regularly were an operations and data committee, a microbiology core unit, a leadership committee, and a publications committee; the first two groups addressed specific operational and technical laboratory issues to move the study forward, respectively, and freed the leadership committee to discuss larger-scale topics related to study progress. The publications committee, which comprised of two co-investigators and one external expert in TB research, steered efforts for the publication of key findings. Additionally, each international study site had a local team, which included principal investigators, a study coordinator, clinicians, and laboratory technicians. Site teams were responsible for recruitment, data collection and transmission, sample processing, and participant follow-up.

#### Website

To share critical information and study documents, a GCDD website was established and utilized regularly throughout the study [22]. This website housed protocols, standard operating procedures, case report forms, meeting agendas and minutes, presentations, publications, contact lists, and reports for the central study management or sites to view, and in some cases, download to provide responses. Users at the sites could upload requested source documents via the website directly to the data coordinating center and were able to access electronic data capture ystems for data entry. Moreover, the website allowed for access levels to be specified so that confidential information was protected from unauthorized users. The GCDD website was managed by the data coordinating center, and will continue to be maintained after the study funding period, allowing for public requests to access to study data.

#### Site visits

Visits to enrollment sites were required by a representative of Family Health International prior to study initiation. The objectives of these visits were to conduct a general assessment of the site and to gather information on its structure and organization, as it pertained to compliance with good clinical practices and other requirements of the National Institutes of Health. Advice was given on strengthening practices to meet good clinical practice requirements. The principal investigator and co-investigators also visited the sites to verify compliance with standard operating procedures and respond to questions or difficulties encountered in executing the study protocol. These preparatory visits occurred before or during the validation phase. During the clinical observation phase, co-investigators visited India and Moldova to monitor study progress.

#### Data management

The University of California, San Diego, Health Services Research Center, a multidisciplinary team of researchers, database managers, and programmers, acted as the data coordinating center, collecting and managing study data. The study also employed a quality assurance manager who worked in collaboration with the data coordinating center to ensure high-quality data throughout the study. All data were stored and managed in a secure SQL database using SQL Server Management Studio, version 2012.

A top priority of this study was to protect subject confidentiality. All data entry systems were password protected and only the clinician enrolling subjects had access to personally identifying information, such as name, date of birth, or contact information. These were used only for follow-up purposes. Clinicians were instructed how to password protect the document containing personally identifying information so that the data managers and programmers were blinded to this information when accessing the laptop. Study personnel were instructed never to document the participants’ names and study identification numbers in the same location on any study document.

#### Data capture systems

Data were gathered by two independent electronic data capture systems: clinical case report forms were entered directly into a password-protected study laptop as clinicians interviewed subjects or gathered medical record information via software designed specifically for this study. This application allowed the user to collect data off-line (a function critical for enrollment in more rural locations where internet connection was not reliable) and then allowed upload of data to the database when a connection became available. This software also enabled users to save their progress until a later time if needed and assigned the unique identifier that the participant and associated samples carried throughout the study.

The second was a web form system designed by the Health Services Research Center for the collection of laboratory case report forms. Study data were gathered from original laboratory result sheets upon test completion and documented on study case report forms. The laboratory technicians then entered this information into web forms and uploaded the data directly to the database, as all central laboratories had reliable internet connections. The system was comprised of 13 individual web forms. Each sent an automated confirmation email to the site coordinator and the data manager whenever forms were uploaded, as verification of database receipt.

#### Remote access

Owing to the physical distance between the data coordinating center and the study sites, provisions were put into place to troubleshoot technical difficulties remotely. The study utilized the web tool Log Me In [23]. This enabled database managers and programmers to access study laptops when they were connected to the internet, allowing identified problems to be resolved promptly, so that enrollment could progress normally.

#### Data quality assurance and control measures

We utilized several front-end quality assurance measures in the electronic data entry form fields to prevent incorrect data from being included in the database. For instance, electronic forms would not accept a duplicate case and date fields would accept only a response in date format (and only current or past dates, local time). Although these types of front-end quality assurance measures existed on almost every field in each form, some data points still required internal validity checks to verify that information between case report forms was consistent. For these, back-end quality control measures were employed, such as date checks of when laboratory procedures were performed; if a date of test completion for a drug susceptibility test was entered as earlier than the sample processing date, the site would be queried, as this was an illogical process of events. Similarly, if a medication stop date was earlier than the start date, the site would be asked to verify and correct the information.

As a final level of quality control, the microbiology core unit reviewed all laboratory data from 100% of enrolled subjects to ensure that results were logical in the context of the patient. This routine also helped to identify trends that would potentially affect study outcomes, such as the need to repeat drug susceptibility tests or the detection of a high rate of smear negative samples. The sites were generally able to execute successfully the protocol. A review by microbiologists vetted the scientific quality of the complex data and allowed data managers to focus on more routine data queries, such as ranges and basic internal validity.

#### Data correction procedures

Data correction was carefully considered as part of the routine data management of the study. If queries by the data manager or quality assurance manager resulted in erroneous data, sites were sent a standard report and asked to respond specifically within the same report or to fill out an electronic data correction form housed on the GCDD website. If a site identified an error in its own data, the same mechanism could be used to report it. As data corrections were sent to the data coordinating center, the data manager reviewed the changes, and an ongoing SQL database query was updated. This query updated the values of a specific case in accordance with the documented error in a new copy of the data table, so that raw data would be preserved. Only these updated tables were used for analysis. While every effort was made to prevent erroneous data before receipt in the database, this could not always prevent inaccuracies. Establishing a procedure early in the study kept the requests manageable.

#### Data monitoring and reporting

The high volume of information collected made it important to monitor study data in real time. We developed progress reports to ensure that sites regularly transmitted data and to keep collaborators informed of study progress. Many of these reports were run weekly, while others were run monthly. Weekly reports included counts of enrollment, defined by the receipt of the first expected post-screening measure (the enrollment interview case report form); counts of laboratory processing forms by subject; and date of last upload for each measure. Further, the length of time between expected upload and actual upload for each case report form were monitored. It was important to assess clinics and laboratories separately as the processes for each type of data collection were expected to take differing lengths of time. Further, timeliness of responses was affected in part by number of staff members available to assist at each site. A standard protocol was utilized for inquiring about overdue data and reports: first, the data manager reported to the clinical manager following two unresponsive requests to the staff member responsible. If still unresponsive, the clinical manager escalated to the principal investigator, who contacted the site principal investigator directly. It was rare for any request to progress this far, but allowing for this possibility ensured that data monitoring issues were handled consistently and in a timely fashion.

In addition to monitoring the time for receipt of case report forms, we developed detailed reports called reconciliation reports. These site-specific reports included a list of all study IDs and which forms were received, overdue, or not yet due. For outstanding forms, it was requested that sites respond monthly with the status of each. Problems observed in timely data collection were followed up by the data manager, quality assurance manager, and clinical manager. Generally, these reports served as a reminder of what follow-up forms were upcoming or overdue, but at times they also informed managers of more serious internal issues, such as a lack of study supplies to complete a test, and therefore, a case report form.

As data were needed for interim analyses, such as sensitivity and specificity, or the time to result of each rapid test, the database was queried and output was provided in a Microsoft Excel spreadsheet and either emailed to the requestor or posted on the GCDD website. Accompanying documentation was supplied on the website throughout the study. A final data table query was written to compute study outcomes that considered multiple fields. This assisted with generating datasets for analysis much more quickly than on an as-needed basis.

Study subjects’ drug susceptibility test results were made available to the treating physicians at each site. Clinicians were carefully informed of the research nature of the test results and that treatment decisions should not be based solely on the results of study tests. All TB treatment that the subjects received was determined and administered by the local TB clinicians. Treatment or treatment recommendations were not provided through the study. It was emphasized that the results obtained were for research purposes only.

## Results and discussion

Detection time to XDR-TB diagnosis remains a challenge. This study design was intended to advance the development and validation of molecular-based technologies (line probe assay and pyrosequencing) and one microbiological method (MODS assay), which have proven success for the rapid detection of MDR-TB in low-resource settings. Geographic and TB strain differences were expected to affect the genetic mutations associated with drug resistance that were observed, making it important to study the molecular basis for drug resistance and evaluate the performance of rapid diagnostic tests for XDR-TB across different clinical settings. This paper discussed the methodologies used to produce data pertinent to these aims and issues encountered were described.

All sites completed the validation procedures successfully. There are potentially useful data and samples available from that phase of the study that may be described in future publications. The validation phase of the study was critical to testing and modifying the methodologies described. One such adjustment was for the required volume of sputum to maintain eligibility: the validation phase began with a minimum 5 ml of sputum on the first visit from each participant; however, it was determined that to complete all laboratory processes, it would be necessary to raise this minimum to 7.5 ml of pooled sputum. Although the minimum volume increased, applying it to the pooled sputum allowed the morning specimen to be included, allowing a greater chance for screened participants to meet this minimum. This did somewhat affect the retention rate for participants: the withdrawal rates due to a lack of 5 ml sputum on the first visit and 7.5 pooled were 0.8% and 3.1%, respectively. Despite the increased withdrawal rate, all subjects who were retained in the study had adequate samples to meet testing requirements, so that resources could be directed appropriately. In a study of this size, working efficiently and evaluating the outcome of all decisions was incredibly important.

In addition, the ability to monitor enrollment and data entry targets in real time was an important feature of the study design. This allowed any problems with achieving enrollment goals to be addressed quickly and kept researchers well informed of sample size as the study progressed. As data were received in the database, multiple levels of data quality checks were put into use throughout the study. These validations and queries at times revealed both systematic and random errors. While these inaccuracies pointed to improvements that could be made in training data collection staff or to data capture systems, it was the job of the operations and data committee to determine which situations required retraining and which would necessitate improvement of systems or quality control measures. Although careful planning went into each of the case report forms, it is recommended that case report forms and electronic data capture field validations be thoroughly examined by clinical and laboratory personnel prior to enrollment so that internally valid data can be captured with minimum burden on data entry staff. Further, changes to data points being collected might occur but should be tested thoroughly, so as to avoid introduction of new errors within the database. All data should be traceable to a source document, so that information can be followed up. The ability to monitor data quality in real time was significant to identification and resolution of problems that could have severely affected the quality of the final data.

Direct communication with enrollment sites and laboratories were also critical to the study’s success. Monthly site-specific teleconferences were implemented with the central leadership committee and all staff at each of the study sites, so that issues specific to the study site could be the primary focus of conversation. These calls allowed the opportunity to discuss details of requests made in either direction and specific data issues needing resolution, and to provide assistance with troubleshooting equipment problems. This teleconference format was highly productive for this study, as it kept all parties accountable for necessary actions and was more efficient than email communications, which had inherent delays due to time zone differences. It also allowed input from all collaborators, giving confidence that all departments involved were considered when decisions were needed. Further, annual webinars included presentations of data to date, site collaboration, and feedback to the central leadership. These regular communications played a vital role in detecting issues and solutions that were not picked up through routine queries and gave sites the opportunity to share experiences with one another.

The diversity of study locations where recruitment took place was crucial for comparative analyses but was not without challenges. Although many circumstances were planned for, such as implementing provisions for physical distance between the coordinating center and study sites, challenges arose that required consideration and decision-making by the central leadership. For instance, India was the only study site where patients routinely carried their own medical records, as opposed to them being stored at the clinic. This affected follow-up, as subjects were initially only expected to return at day 2 and week 52 for follow-up visits. While this remained the case in Moldova and South Africa, subjects from the India site were asked to return to the clinic 30 days after enrollment to supply chart review information, causing difficulty in obtaining this follow-up measure as quickly as the other two sites, where medical records were stored at the clinic. To improve the success rates of follow-up, it was determined that this site could collect the 30-day data by telephone, by having the subjects read the information from the record. Additionally, the Moldova and South Africa study sites did not have the same patient volumes as the India site, so recruitment was at a slower pace. These needed to be accounted for in recruitment targets. Each site also observed unique holidays and clinic closures, which affected the pace of recruitment. It is recommended that access to medical records, patient volume, and local schedules be carefully considered prior to study launch so that they can be accommodated.

## Conclusions

In summary, we have documented the methodology used in a global multi-site DR-TB study for the advancement of diagnostic tools. By conducting a large prospective study, which captured epidemiological, clinical, and biological data, we have produced a high-quality and unique dataset, which will be beneficial for analyzing study aims as well as answering future DR-TB research questions. Analyses and main findings of the GCDD study are forthcoming. We will also continue to analyze the repository of isolates and DNA gathered from this study, as they are an invaluable resource to evaluate new diagnostic devices as they become available. Reduction in detection time for XDR-TB would be a major public health success, as it would allow for improved treatment and more successful patient outcomes. Executing successful trials is critical in assessment of these reductions in highly variable populations.

## Abbreviations

DR-TB: drug-resistant tuberculosis
GCDD: Global Consortium for Drug-resistant Tuberculosis Diagnostics
MDR-TB: multidrug-resistant tuberculosis
MGIT: mycobacterial growth indicator tube
MODS: microscopic observation drug susceptibility
PCR: polymerase chain reaction
TB: tuberculosis
WHO: World Health Organization
XDR-TB: extensively drug-resistant tuberculosis.

## References

[1] World Health Organization: **Global Tuberculosis Report 2013.** Geneva; 2013. [http://apps.who.int/iris/bitstream/10665/91355/1/9789241564656_eng.pdf]

[2] Dye C, Williams BG (2010). The population dynamics and control of tuberculosis. Science.

[3] Dharmadhikari AS, Basaraba RJ, Van Der Walt ML, Weyer K, Mphahlele M, Venter K, Jensen PA, First MW, Parsons S, McMurray DN, Orme IM, Nardel EA (2011). Natural infection of guinea pigs exposed to patients with highly drug-resistant tuberculosis. Tuberculosis.

[4] Escombe AR, Moore DAJ, Gilman RH, Pan W, Navincopa M, Ticona E, Martinez C, Caviedes L, Sheen P, Gonzalez A, Noakes CJ, Friedland JS, Evans CA (2008). The infectiousness of tuberculosis patients coinfected with HIV. PLoS Med.

[5] Fauci AS (2008). Multidrug-resistant and extensively drug-resistant tuberculosis: the National Institute of Allergy and Infectious Diseases Research agenda and recommendations for priority research. J Infect Dis.

[6] Rodwell TC, Valafar F, Douglas J, Qian L, Garfein RS, Chawla A, Torres J, Zadorozhny V, Kim MS, Hoshide M, Catanzaro D, Jackson L, Lin G, Desmond E, Rodrigues C, Eisenach K, Victor TC, Ismail N, Crudu V, Gler MT, Catanzaro A (2014). Predicting extensively drug-resistant *Mycobacterium tuberculosis* phenotypes with genetic mutations. J Clin Microbiol.

[7] Almeida D, Rodrigues C, Udwadia ZF, Lalvani A, Gothi GD, Mehta P, Mehta A (2003). Incidence of multidrug-resistant tuberculosis in urban and rural India and implications for prevention. Clin Infect Dis.

[8] Jenkins HE, Plesca V, Ciobanu A, Crudu V, Galusca I, Soltan V, Serbulenco A, Zignol M, Dadu A, Dara M, Cohen T (2013). Assessing spatial heterogeneity of multidrug-resistant tuberculosis in a high-burden country. Eur Respir J.

[9] World Health Organization: **Global Tuberculosis Report 2012.** Geneva; 2012 [http://apps.who.int/iris/bitstream/10665/75938/1/9789241564502_eng.pdf]

[10] World Health Organization: **Laboratory Services in Tuberculosis Control, Part III: Culture.** Geneva; 1998. [http://whqlibdoc.who.int/hq/1998/WHO_TB_98.258_(part3).pdf]

[11] Lin SYG, Rodwell TC, Victor TC, Rider EC, Pham L, Catanzaro A, Desmond EP (2014). Pyrosequencing for rapid detection of extensively drug-resistant *Mycobacterium tuberculosis* in clinical isolates and clinical specimens. J Clin Microbiol.

[12] World Health Organization: **Policy Guidance on Drug-Susceptibility Testing (DST) of Second-Line Antituberculosis Drugs.** Geneva; 2008. [http://whqlibdoc.who.int/hq/2008/WHO_HTM_TB_2008.392_eng.pdf?ua=1] 26290924

[13] Rodrigues C, Jani J, Shenai S, Thakkar P, Siddiqi S, Mehta A (2008). Drug susceptibility testing of *Mycobacterium tuberculosis* against second-line drugs using the Bactec MGIT 960 system. Int J Tuberc Lung Dis.

[14] Lin SYG, Desmond E, Bonato D, Gross W, Siddiqi S (2009). Multicenter evaluation of Bactec MGIT 960 system for second-line drug susceptibility testing of *Mycobacterium tuberculosis* complex. J Clin Microbiol.

[15] Barnard M, Albert H, Coetzee G, O’Brien R, Bosman ME (2008). Rapid molecular screening for multidrug-resistant tuberculosis in a high-volume public health laboratory in South Africa. Am J Respir Crit Care Med.

[16] Brossier F, Veziris N, Aubry A, Jarlier V, Soudakoff W (2010). Detection by GenoType MTBDR*sl* test of complex mechanisms of resistance to second-line drugs and ethambutol in multidrug-resistant Mycobacterium tuberculosis complex isolates. J Clin Microbiol.

[17] Kiet VS, Lan NT, An DD, Dung NH, Hoa DV, van Vinh CN (2010). Evaluation of the MTBDR*sl* test for detection of second-line-drug resistance in *Mycobacterium tuberculosis*. J Clin Microbiol.

[18] Diggle MA, Clarke SC (2004). Pyrosequencing^TM^. Mol Biotechnol.

[19] Bravo LTC, Tuohy MJ, Ang C, Destura RV, Mendoza M, Procop GW, Gordon SM, Hall GS, Shrestha NK (2009). Pyrosequencing for rapid detection of *Mycobacterium tuberculosis* resistance to rifampin, isoniazid, and fluoroquinolones. J Clin Microbiol.

[20] Moore DAJ, Evans CAW, Gilman RH, Caviedes L, Coronel J, Vivar A, Sanchez E, Piñedo Y, Saravia JC, Salazar C, Oberhelman R, Hollm-Delgado MG, LaChira D, Escombe R, Friedland JS (2006). Microscopic-observation drug-susceptibility assay for the diagnosis of TB. N Engl J Med.

[21] Trollip AP, Moore D, Coronel J, Caviedez L, Klages S, Victor T, Romancenco E, Crudu V, Ajbani K, Vineet VP, Rodrigues C, Jackson RL, Eisenach K, Garfein RS, Rodwell TC, Desmond E, Groessl EJ, Ganiats TG, Catanzaro A (2014). Second-line drug susceptibility breakpoints for *Mycobacterium tuberculosis* using the MODS assay. Int J Tuberc Lung Dis.

[22] **Global Consortium for Drug-resistant Tuberculosis Diagnostics** [http://gcdd.ucsd.edu]

[23] **LogMeIn** [https://secure.logmein.com/]

